# Anticancer Chemotherapy and it's Anaesthetic Implications (Current Concepts)

**Published:** 2009-02

**Authors:** R P Gehdoo

**Affiliations:** Professor, Tata Memorial Hospital, Mumbai

**Keywords:** Anticancer chemotherapy drugs, Systemic effects, Implications to anaesthetic management

## Abstract

**Summary:**

Many a times, cancer patients undergo chemotherapy before being subjected for surgery. Such patients pose some serious interactions and complications during the anaesthetic management. So, it is very important to know such interactions, and problems in advance for a smoother and uncomplicated management of anaesthesia. Herewith, a detailed review of this problem is discussed along with the current concepts and solutions.

## Introduction

Cancer is treatable if detected early. Cancer is the second leading cause of death in United States^1^. It is a complex matter having special considerations. Hence, cancer patients deserve special anaesthetic considerations. It requires a very close cooperation among surgeon, anaesthesiologist, and referring physician to assure the conduct of surgical procedures on the patient with cancer with maximal safety.

Chemotherapy forms an important aspect of cancer treatment. With an increased number of patients surviving for a longer period of time, a number of patients, who have received chemotherapy, may be subjected to elective and emergency surgery, therefore it is essential to know the effects of the chemotherapeutic agents on normal organ systems. The toxicity of cancer chemotherapy drugs and their relevance to perioperative anaesthesia management relates to the specific agents used, their cumulative dosage, and drug toxicity etc. The most common toxicities to chemotherapeutic agents include cardiac, pulmonary, hematologic, bone marrow, and gastrointestinal effects. Coagulopathies, thrombocytopenia, and anaemia with ulceration and bleeding of the gastrointestinal tract may often occur.^2^.

Table 1 summarizes various cancer chemotherapy agents, their toxicities, and their relevance to the anaesthesiologist^3^. Of particular importance to the anaesthesiologist in the peri-operative period are the effects of chemotherapeutic agents on the cardio-pulmonary system as well as the other organ systems which is discussed underneath in details.

**Table 1:** Common complications associated with cancer chemotherapy agents

System toxicity	Chemotherapeutic agents
Cardiac toxicity	Busulphan, cisplatin, cyclophosphamide, daunorubucin, 5-fluorouracil
Pulmonarytoxicity	Methotrexate, bleomycin, busulphan, cyclophosphamide, cytarabine, carmustine
Renal toxicity	Methotrexate, L-asparginase, carboplatin, ifosfamide, mitomycin-C
Hepatic toxicity	Actinomycin D, methotrexate, androgens, L-asparginase, busulphan, cisplatinum, azathiopine
CNS toxicity	Methotrexate, cisplatin, interferon, hydroxyurea, procarbazine, vincristine
SIADH secretion	Cyclophosphamide, vincristine

The effects and problems occurring because of anticancer chemotherapy and it's implications on the anaesthetic management can be discussed under the following headings-

- Cardiovasculareffects and complications following chemotherapy
- Pulmonary effects and complications following chemotherapy
- Other systems affected by chemotherapy (Hepato-renal, CNS, Haemopoetic system)
- Miscellaneous important complications

## A) Cardiovascular effects and complications following chemotherapy

Cancer patients receive a series of chemotherapeutic agents that may adversely affect the heart.^4^–^6^

Anthracyclines; i.e. doxorubicin (adriamycin), daunorubicin, and epirubicinare the commonest agents implicated in the development of cardiac toxicity after cancer chemotherapy. Cardiac toxicity can manifest at various times during and following the course of chemotherapy, three types depending on their appearance in relation to timing of therapy, have been identified.

Anthracycline agents may impair myocardial contractility.^7^. Similarly, patients receiving mitoxantrone at a total dose of more than 140 mg/m^2^ can suffer congestive heart failure and anthracycline-induced cardiomyopathy. Another agent known to cause myocardial tissue injury is cyclophosphamide^8^

A cyclophosphamide dose range of more than 120mg.kg^−1^ over 2 days can result in severe congestive heart failure and haemorrhagic myocarditis, pericarditis, and necrosis. Patients receiving busulfan in conventional oral daily dosage may suffer endocardial fibrosis, with signs and symptoms of constrictive cardiomyopathy.^9^,^10^

Patients with preexisting cardiac disease receiving interferon in conventional doses may have exacerbations of their underlying illness. More recently, the use of mitomycin for extended periods of time and dosages has been shown to produce myocardial damage.^11^

Previous treatment with anthracyclines may enhance the myocardial depressive effect of anaesthetics even in patients with normal resting cardiac function.^12^ The preoperative and anaesthetic assessment of the patients who have received these above mentioned agents may require 2D-echocardiogram or nuclear medicine studies. Such studies permit precise measurement of the left ventricular ejection fraction and detection of regional and global myocardial dysfunction. Where congestive failure is discovered, the physician will have to treat it preoperatively.

In addition to the above side effects, anthracycline agents can cause dysrhythmias unrelated to the cumulative dose.^13^. Such dysrhythmias may occur hours or even days after administration. Commonly observed dysrhythmias include supraventricular tachycardia, complete heart blocks, and ventricular tachycardia. In addition, doxorubicin may prolong the QT interva1.^14^

In recent years, it has been observed that paclitaxel, when given in combination with cisplatinum, may also produce ventricular tachycardia^15^

### Acute and Subacute cardiotoxicity:

It can occur immediately after a single dose or a course of anthracycline therapy. Acutetoxicity commonly (40%) takes the form of ECG changes such as nonspecific ST-T changes, decreased QRS voltage, and QT prolongation. Decreased R wave amplitude has been thought by some to signal development of chronic cardiomyopathy later, though it is not proved. Sinus tachycardiais the most common rhythm disturbance but avariety of arrhythmias, including ventricular, supraventricular, and junctional tachycardia, have been reported. Atrioventricular and bundle-branch block have also been seen^16^.

These changes occur at all dose intervals and except for decreased QRS voltage, resolve 1 to 2 months after cessation of the therapy. Sudden death may also occur, due to ventricular fibrillation. Rare cases of subacute cardiotoxicity resulting in acute failure of the left ventricle, pericarditis or a fatal pericarditis-myocarditis syndrome, particularly in children, have been reported^17^. If these patients recover they should not receive further treatment with anthracyclines. In elderly patients with preexisting heart disease, congestive heart failure can occur, which is generally transient and responds to normal medical management.

### Chronic or late cardiotoxicity:

Chronic cardiotoxicity after anthracyclines classically takes the form of cardiomyopathy. CXR review may reveal cardiomegaly. ECG changes occur with these agents and includenon-specific ST-and T-wave changes, prematureatrial and ventricular contractions, sinus tachycardia and low-voltage QRS complexes.^18^ Anthracycline cardiotoxicity is a cumulative dose related phenomenon. The incidence of congestive heart failure secondary to anthracycline induced cardiotoxicity increases with dose. Praga et al^19^ reported that an average incidence of 7% at 550 mg/m^2^, 15% at 600 mg/m^2^, and 35% at 700 mg/m^2^. At total doses^20^ less than 400 mg/m^2^ the incidence of CHF is 0.14%. The rapid increasein incidence of CHF after a dose of 550 mg/m^2^ has made it a popular empiric-limiting dose for doxorubicin-induced cardiotoxicity.

### Late onset cardiotoxicity:

Several recent studies, extensively reviewed elsewhere^21^ have reported occultventricular dysfunction, heart failure and arrhythmias occurring in previously asymptomatic patients more than a year after anthracycline therapy.^22^,^23^. It is postulated that doxorubicin can cause subclinical myocardial injury during pre-adolescent years and this in later years retards appropriate growth of the myocardium during growth spurt.

### Pathogenesis ofanthracycline cardiotoxicity:

The anthracycline antibiotics react with cytochrome P-450 reductase in the presence of reduced nicotinamide adenine dinucleotide phosphateto form semiquinone radical intermediates, which in turn can react with oxygen to form superoxide anion radicals. These can generate both hydrogen peroxide and hydroxylradicals, which are highly destructive to cells thus causing myofibrillarlysis, cytoplasmic vacuolization, and degeneration of nuclei and mitochondria in the myocytes. Severe myocyte damage results in decreased myocardial contractility and CHF.

### Risk factors for development of anthracycline cardiotoxicity:

Apart from the total dose, patients who have received high dose radiation to the mediastinum and those who are on concurrent cyclophosphamide therapy are particularly susceptible to this cardiomyopathy. The other risk factors are extremes of age, prior ischaemic heart disease, hypertension, valvular heart disease and liver diseases. The risk involved at a cumulative dose in the range of 300-450 mg/m^2^ is about 1-10%, while doses higher than this invites a risk of>30%.

### Investigations for the detection of anthracycline cardiotoxicity:

The best currently available noninvasive method for assessing cardiac function is radionucleide angio-cardiography. The commonly studied parameters with radionucleide studies are left ventricular ejection fraction (LVEF). A decrease in LVEF to less than 45% is considered to indicate anthracycline-induced cardiotoxicity. 2-Dechocardiography is a non-invasive method of cardiac valuation. Diastolic dysfunction on echocardiogram may represent an earlier manifestation of anthracycline toxicity. The newer noninvasive methods to know the actual myocardial damage are by using imaging with monoclonal indium–111–antimyosin antibodies. These antibodies bind to the exposed myosin in the necrosed myocardial cells. A diffuse uptake on imaging indicates a generalised process such as anthracycline cardiomyopathy; a focal uptake will suggest local pathology such as myocardial infarct.^24^

Other chemotherapeutic agents also have adverse cardiac effects, which are important for the anaesthetist to know. Table 2 summarizes them as follows:-

**Table 2:** Other chemotherapeutic agents having adverse cardiac effects.

Agent	Cardiovascular effect
Cyclophosphamide	Fulminant CHF secondary to hemorrhagic myocarditis
	Acute Pericarditis with effusion
	Risk increased with dose > 200mg/m^2^ & anthracycline combination
Bleomycin	Acute pericarditis
5 – Fluorouracil	Coronary insufficiency presenting as angina / myocardial infarct due to coronary spasm
Paclitaxel and docetaxel	Asymptomatic bradycardia, severe brady & tachyarrhythmias including ventricular fibrillation & asystole, conduction disorders myocardial ischaemia, infarction, risk increased with concomitant cisplatinum therapy peripheral edema due to fluid retention (docetaxel)

### Problems with anaesthesia management:-

An appropriate anaesthetic plan including the invasive monitoring techniques hinges on thorough pre-operative assessment. Invasive arterial blood pressure recordings and apulmonary artery catheterization may be necessary if significant myocardial impairment is present. Anthracycline treated patients under anaesthesia can develop acute intraoperative left ventricular failure refractory to β- adrenergic receptor agonists. Amrinone and sulmazole are the new class of cardiotonics with inotropic drugs useful in such conditions.

## B) Pulmonary effects and complications of cancer chemotherapy

Cancer patients commonly suffer pulmonary complications. 75% to 90% of pulmonary complications are secondary to infection. The cancer patient can suffer infectious complications secondary to chemotherapy (e.g., Bleomycin), thoracic radiation, and multiple pulmonary resections.^25^

Pulmonary complications are a significant problem; respiratory failure in cancer patients requiring assisted mechanical ventilation is associated with a 75% mortality rate.^26^–^28^ In patients with systemic cancer, the differential diagnosis of pulmonary infiltrates seen on a routine chest radiograph is extensive; there are many causes for such infiltrates.^29^–^31^

Administration of several chemotherapeutic agents, such as busulfan, cyclophosphamide, paclitaxel, etc, can lead to pulmonary complications. Bleomycin, an antitumour agent, is the foremost of these in producing lung damage.

### Several patterns of pulmonary toxicity produced by bleomycin have been described:

1. Dose dependent interstitial pneumonitis progressing to chronic fibrosis
2. An acute hypersensitivity pneumonitis with peripheral eraleosinophilia resembling eosinophilic pneumonia.
3. An acute chest pain syndrome.
4. A bronchitis obliterans with organising pneumonia.
5. Pulmonary veno-occlusive disease.

About 0-40% patients are reported^32^ to develop pulmonary toxicity, 11-30% patients will have non-lethal pulmonary fibrosis and the mortality associated with bleomycin toxicity will range from 2-10%. Progressive interstitial pneumonitis and fibrosis is the most common pattern of bleomycin lung injury. Symptoms generally occur between 4 to 10 weeks after bleomycin therapy, however in about 20% patients with radiographic and histological features of bleomycin toxicity may be present without any clinical symptoms. The risk factors for bleomycin pulmonary toxicity are old age, acumulative dose >400-450 U, poor pulmonary reserves, radiotherapy, uraemia, higher inspired oxygen concentrations, and concomitantly administered other anticancer drugs.

### Mechanisms of pulmonary toxicity:

Though the threshold dose level for the development of pulmonary disease is in the range of 400 to 450mg, fatal pulmonary fibrosis has been reported with doses as low as 50mg. The mechanism of pulmonary toxicity associated with the use of bleomycin, is probably due to direct cytotoxicity and in patients receiving bleomycin, type I pneumocytes are replaced by type II pneumocytes. Continued exposure to bleomycin prevents reversion of type II to type I pneumocytes and further leads to meta-plasia of the type II cells to cuboidal epithelium. Further exposure prevents effective repair and fibrosblasts and macrophages migrate into the interstitium and the alveoli. Ultimately pulmonary fibrosis results. One proposed mechanism for bleomycin toxicity involves the production of superoxide and other free radical moieties, which then cleave nuclear DNA. The production of these highly oxidizing radicals might be increased by the inspiration of fortified concentrations of oxygen.

### Clinical presentation:

The lesions seen frequently are in the lower lobes and sub pleural areas and chest X-ray shows bilateral basal and peri-hilar infiltrates with fibrosis. The first signs and symptoms of toxicity are fever, cough, dyspnoea and bibasilar rhonchi and rales, which may progress to exertional dyspnoea with mild X-ray changes and a normal resting PaO_2_ or a severe form of hypoxia at rest. The earliest detection of pulmonary fibrosis may be achieved through the serial evaluation of pulmonary function. Sequential measurement of carbon monoxide diffusion capacity (DLCO) may indicate the presence of occult pulmonary changes. Arterial hypoxemia is commonly found and spirometry reveals decreased lung volumes compatible with restrictive lung disease. Regression or amelioration of the toxic pulmonary pathology may occur with immediate cessation of therapy. Steroid therapy has been found to be effective in some cases.

Occasionally, it may manifest itself as noncardiogenic pulmonary edema, chronic pneumonitis and fibrosis, and hypersensitivity pneumonitis.^33^

### Hyperoxia & Bleomycin:

Of utmost importance to the anaesthesio logist is the debate about the amount of oxygen to be administered to a patient coming up for surgery after being given bleomycin. The debate was sparked off by a land-mark report by Goldiner etal^34^. They described 5 patients undergoing surgery after receiving bleomycin, given >39% oxygen intraoperatively, developed ARDS and died. Subsequent 13 patients, given > 25% oxygen, survived the surgery without pulmonary complications^35^. This need to restrict inspired oxygen concentration was subsequently questioned by La Mantia et al, who reported a series of 16 patients with uncomplicated perioperative period in spite of receiving high FiO_2_ (>0.41) intraoperatively^36^

A recent study by Donat et al^37^ evaluating 77 patients undergoing 97 extensive resection procedures after receiving bleomycin seems to have solved the issue. They found that, though the inspired concentration of oxygen was > 40%, and 25% patients did develop minor pulmonary complications; none of them developed ARDS or died. The authors concluded that perioperative oxygen restriction is not necessary and a meticulous perioperative fluid balance including transfusions as a significant predictor of postoperative pulmonary morbidity. They also noted that the duration of surgery and post-chemotherapy forced vital capacity are significant predictive factors of procedure related pulmonary morbidity. On the basis of available data it seems prudent to reduce the concentration of inspired oxygen to the lowest level to maintain SpO_2_ > 90%. Intraoperative monitoring is the key to safe administration of oxygen to these patients. Arterial blood gas analysis should be performed by an indwelling arterial cannula or intermittent sampling. The judicious use of intraoperative PEEP to enhance oxygenation and the postoperative use of rigorous physiotherapy to treat ventilation-perfusion abnormalities may be preferable to the use of enriched oxygen concentrations. Fluid balance is another important factor in predicting pulmonary morbidityin-patients receiving bleomycin. Conservative fluid management is important; use of colloids is beneficial as compared to crystalloid.

Other chemotherapeutic agents also have adverse pulmonary effects, which are very vital for the anesthetist to understand. Table 3 summarizes them as follows:-

**Table 3:** Other chemotherapy agents having adverse pulmonary effects

Agents	Incidence	Description
Busulfan	4-10%	Pulmonary fibrosis, Pulmonary alveolar lipoproteinosis
Cyclophosphamide	<2%	pneumonitis with or without fibrosis
Mitomycin	<10%	Similar to bleomycin
Cytosine arabinoside	5 - 32%	Non-cardiogenic pulmonary edema with or without pleural effusion
Methotrexate	7%	Hypersensitivity pneumonitis like picture or
		Non-cardiogenic pulmonary edema or
		Pulmonary fibrosis or
		Pleurisy with acute chest pain

## C) Effects of cancer chemotherapy agents on hepato-renal, and CNS system

### i) Renal complications:-

**Cisplatinum,** a commonly used anticancer drug has been found to produce toxic effects like nephrotoxicity, myelosuppression, neuropathy in stocking and glove distribution, auditory and visual impairment. The dose-limiting factor for single agent use, however, is nephrotoxicity. 30% of patients receiving cisplatinum will develop nephrotoxicity, especially if the hydration is not properly controlled. It causes coagulation necrosis of proximal and distal renal tubular epithelial cells and in the collecting ducts leading to are duction in the renal blood flow and glomerular filtration rate (GFR). Cisplatinum leads to wasting of magnesium and potassium. A single dose of 2mg/kg or 50-75mg/m^2^ will produce nephrotoxicity in 25-30% of patients^38^.

Acute renal failure can result within 24 hours of administration of a single dose of cisplatinum. The long-term effects were reported by Fjeldberg et al^39^ who found a 12.5% reduction of GFR after 16 to 52 months after administration of cisplatinum. Proper hydration with forced diuresis seems to decrease the incidence of renal toxicity by reducing the concentration of cisplatinum in renal tubules and the amount of time it remains in contact with renal tubules. Use of normal saline is particularly beneficial as high chloride concentrations in the tubules inhibit the hydrolysis of cisplatinum. The renal toxicity may be accentuated if the patient receives aminoglycosides concomitantly. The newer analogues of cisplatinum, such as carboplatinum and oxaloplatinum are less nephrotoxic with equal efficacy in controlling the malignancy.

Methotrexate causes the acute nephrotoxicity as a result of its intratubular precipitation^40^.

Other chemotherapeutic agents also have adverse renal effects are summarized in the Table 4 as follows:-

**Table 4:** Other chemotherapy agents having adverse nephrotoxic effects

Drug	Description
Mitomycin	Chronic progressive rise in serum creatinine to microangiopathic hemolytic anemia
Methotrexate	Physical effect because of precipitation of drug in renal tubules
Ifosphamide	Acute tubular necrosis & renal failure

### ii) CNS complications:-

**Vinca alkaloids** were the first anticancer drugs found to have neurotoxiceffects. Vincristine is probably the only drug whose dose limiting toxicityis neurotoxicity. It can affect the central, peripheral or the autonomic nervous systems. Peripheral neuropathies present as peripheral paresthesias with depression of deep tendon reflexes. The paresthesias progress proximally with therapy. Motor dysfunction and gait disorders can occur. Vincristine,^41^ vinblastine, procarbazine, cisplatinum^42^ all can cause a toxic neuropathy with paresthesia, loss of deep tendon reflexes and muscle weakness.

Autonomic neuropathy with orthostatic hypotension is a rare concomitant of neoplasia^43^. Cranial nerve effects may manifest as opthalmoplegia and facial palsy. Autonomic neuropathy can present asorthostatic hypotension, erectile dysfunction, constipation, difficulty in micturition, bladder atony, etc.

**Cisplatinum,** along with its effects on the kidney also affects the nervous system. 50% patients receiving cisplatinum will display neurotoxicity depending on dose and treatment duration. It generally takes the form of paresthesias. Continued treatment will lead to loss of deep tendon reflexes, vibration sense and sensory ataxia.

As far as regional anaesthesia is concerned, one should be aware that in a considerable percentage of patients a sub-clinical, unrecognized neuropathy may be present in patients with previous cisplatinum chemotherapy. Recently, a diffuse brachial plexopathy after interscalene blockade has been reported in a patient receiving cisplatinum chemotherapy. Thus, if regional anaesthesia is contemplated, a detailed pre-operative neurological examination and careful assessment of the risks and benefits is warranted.^44^

Other chemotherapeutic agents also have adverse CNS effects are enumerated in the Table 5 as follows:-

**Table 5:** Other chemotherapy agents having adverse neurotoxic effects

Drug	Incidence of toxicity	Description
Cytarabine	15–37%	Cerebellar dysfunction, peripheral neuropathy, seizures, encephalopathies, myelopathy, pseudobulbar palsy
Ifosphamide	0–10%	Cerebellar dysfunction, hemiparesis, coma, extrapyramidal abnormalities
5-Fluouracil	0-5%	Cerebellar dysfunction, multifocal leukoencephalopathy
Methotrexate	0-2%	Meningeal irritation, transient paraparesis, encephalopathy
Paclitaxel	50 – 70% (high dose)	Peripheral neuropathy, autonomic neuropathy
Procarbazine	---	Cerebral effects: lethargy, depression to psychosis, peripheral neuropathy

### iii) Hepatic complications:-

Hepatocellular dysfunction is manifested as raised serum enzymes, fatty infiltration of liver and cholestasis, due to direct toxic effect of the drug or it's metabolite. L-asparginase and cytarabine are most commonly implicated agents in hepatocellular dysfunction. A decreased synthetic function with low proteins and coagulation abnormalities may be seen. Ascites, painful hepatomegaly, and encephalopathy may result after administration of cytarabine, cyclophosphamide, mitomycin, etc.

## D) Miscellaneous adverse effects of cancer chemotherapy agents:

### i) Haematological complications:-

Bone marrow function in cancer patients may be disturbed by primary bone marrow disorders (e.g., leukemia), bony metastases (e.g., from breast cancer), as well as myelosuppressive chemotherapy. The production of any or all blood elements may be impaired. There is dysfunctional coagulation. The PT and PTT are shortened. There is increase in factor I, V, VIII, IX, XI and FDP. There is reduced survival of the platelets and the decreased antithrombin III activity. There are no prospective trials to date that establish the minimal platelet count necessary to prevent bleeding with specific procedures. Some investigators have maintained a minimal level of 50,000 platelets per microliter in the intraoperative and postoperative period. Correction of other coagulation disturbances is important before undertaking surgical intervention in the thrombocytopenic patient. In view of these findings, a close cooperation among the surgeon, anaesthesiologist, and hematologistis required for optimal management and maximal safety.^45^

Myelo-suppression caused by all the chemotherapeutic agents is partially or completely reversible within 1 to 6 weeks of termination of therapy.

### ii) Syndrome of inappropriate antidiuretic hormone secretion (SIADH):-

Another metabolic abnormality in patients with cancer like lung, pancreas-adeno-carcinoma, duodenum, thymoma, mesothelioma, leukaemia, hodgkin, reticulum cell sarcoma, is SIADH, which occurs in 1% to2% of cancer patients.

Some drugs, such as vasopressin, carbamazepine, oxytocin, vincristine, vinblastine, cyclophosphamide, phenothizianes, tricyclic antidepressant agents, narcotics, and monoamine oxidase inhibitors, can also induce SIADH.^46^

### iii) Steroid administration:

The oncology patient often has a history of exogenous glucocorticoid administration as part of a chemotherapy regimen. The physician at the time of pre-operative evaluation has to decide on the use and the amount of stress steroid coverage. The patient who has received ≥2 weeks of glucocorticoids within the past year is considered at risk for adrenal suppression. However, many of these patients are capable of a normal stress response. The corticotrophin (ACTH) stimulation test is the definitive test to identify adrenal suppression.

### iv) Tumorlysis syndrome:-

Another frequently seen complication in cancer patients is the tumor lysis syndrome.^47^ Chemotherapy induces rapid tumor cell lysis in patients with a large malignant cell burden over an exquisitely sensitive tumor^48^,^49^ This classically occurs in patients with Burkitt's lymphoma, non-Hodgkin's lymphomas, acute lymphoblastic and nonlymphoblastic leukemias, and chronic myelogenous leukemia. In addition, it may also occur continuously in patients with lymphomas and leukemia following treatment with chemotherapy, radiation, glucocorticoids, tamoxifen, or interferon. The clinical mani-festations of this syndrome are related to the metabolic abnormalities.

In those patients with suspected tumor lysis syndrome or for those patients who receive chemotherapeutic agents likely to induce the syndrome, prevention is the mainstay of treatment. To prevent the development of acute renal failure, patients who are to undergo treatment for malignancies should receive vigorous intravenous hydration, often with diuretics or renal doses of dopamine to ensure adequate urine output.

### v) Chemotherapy and wound healing:-

The outcome of surgical procedures may be affected by the wound-healing impairment caused by antineoplastic agents used to treat the underlying tumor. The neutropenia that accompanies some chemotherapy within 7 to 10 days of administration can interfere with the early phases of wound healing. Most patients with WBC count 500/mm^3^ have no adverse effects of leukopenia on surgical wound healing. Chronic anemia also has little effect on surgical wound healing^50^.

The effects of chemotherapy directly on wound healing depend on doseand the timing of drug administration relative to creation of the wound. A high incidence of wound complications has been reported in women undergoing mastectomy after receiving preoperative chemotherapy and radiation. Bleomycin has not been associated with increased wound complications.

## Anaesthetic considerations for patients after chemotherapy

The interaction between an anaesthesiologist and a cancer patient starts with a preoperative visit for a surgical procedure. The goals of such a preoperative visit would be as follows:-

- To optimize patient's physical status.
- To assess effects of cancer and cancer therapies (chemotherapy, radiotherapy and surgery) on patient.

Some of the important features and care before planning anaesthesia in such a chemotherapy received patient are as follows–

- In pre-anaesthetic checkup, one must obtain a pertinent, comprehensive past medical history, preexisting condition prior to surgery and anaesthesia, medications, allergies, family history and a complete systemic examination. The anaesthesiologist's role in pre-operative evaluation and preparation of the surgical patient and intraoperative and postoperative management is of great importance. This begins with a thorough history and physical examination.
- Routine clinical tests like complete blood count, urine analysis, serum electrolytes, fasting blood sugar, serum BUN, pulmonary function tests, PaO_2_ and PaCO_2_ contents by arterial blood gas analysis, serum osmolality, bilirubin, creatinine, amylase, liver function tests, chest X-ray and ECG are mandatory.^51^ Awareness of the side effects of the various chemotherapeutic agents enables other appropriate investigations to be carried out and institution of corrective measures when possible will ensure a well-prepared patient.
- Immuno-suppresion occurs with the use of all the alkylating agents. Meticulous attention must be given to aseptic techniques in the perioperative period in order to avoid potentially lethal iatrogenic infection.
- Pneumonitis and pulmonary fibrosis may be induced by many of the chemotherapeutic agents. History or symptoms suggestive of exertional dyspnea or dyspnea at rest should alert the physician to this problem. In addition to chest X-ray, arterial blood gases are necessary. Pulmonary function tests including arterial blood gas, spirometry, and carbon monoxide diffusing capacity should be evaluated. Findings compatible with interstitial fibrosis include increased alveolararterial gradient, restrictive lung disease, and decreased carbon monoxide diffusing capacity.^52^ Patients who had a bleomycin therapy should not receive high inspired oxygen concentrations and that colloid rather than crystalloid replacement should be preferred both during and after surgery. Ventilator support should be anticipated in the postoperative period.
- Cardiotoxicity may occur in-patients who have received anthracyclines. The ECG may reveal diminution of the QRS voltage, systolic timed interval may be increased, and ejection fraction as well as fractional shortening may be decreased. Congestive heart failure is treated using diuretics, digitalis and oxygen. Operating and recovery room monitoring should include ECG, urinary output, central venous pressure and when feasible pulmonary arterial and wedge pressures. Huetteman and colleagues^12^ showed that previous treatment with anthracycline might enhance the myocardial depressant effects of anaesthetics even in individuals with healthy cardiac function at rest.
- Hepatotoxicity may occur with the use of most of the anticancer drugs. Anaesthetic drugs incriminated, as causing liver damage should not be administered. Busulfan, methotrexate, cisplatinum and others may cause nephrotoxicity. Balanced electrolyte solutions started the evening before surgery will aid in maintaining optimal renal flow and glomerular filtration. Potentially nephrotoxic drugs should be avoided.
- The effects of cyclophosphamide, a pseudo-cholinesterase inhibitor, could last for 3–4 weeks from the end of its use, and Zsigmond and Robins^53^ argued that this occurrence might justify or explain the recognised hazard because of the drug's interaction with suxamethonium (a depolarising muscle relaxant, metabolised by pseudocholinesterase), inducing a risk of protracted postoperative apnoea.
- Negative interactions between methotrexate and non-steroidal anti-inflammatory drugs (NSAIDs) are well known. Although the mechanism of this interaction is not completely clear, NSAIDS are known to reduce the excretion of methotrexate. A competition for receptor sites at renal tubular excretion has been postulated but the mechanism is not yet understood; this interaction might result in potentially fatal side-effects for patients.^54^
- Central and autonomic nervous system toxicity and peripheral neuropathies occur with vincristine, cisplatinum and others. Therefore regional anaesthesia is contraindicated. The preoperative state of sensorium and neurologic deficits should be documented. Anti-cholinesterase effects of alkylating agents are significant. Reduction of dosage of succinylcholine is indicated to prevent prolonged respiratory depression. Monoamine oxidase like inhibition may occur with the administration of procarbazine. Because of synergistic action barbiturates, antihistaminic, phenothiazines, narcotics and tricyclic antidepressants should be used with caution. Diarrhea is a side effect of many of the anti-cancer drugs. Attendant serum electrolyte and fluid abnormalities should be corrected before surgery and also in the postoperative period.

The cancer patient like any other high risk patients requiring anaesthesia deserves a special care and considerations. A growing number of patients undergo surgical procedures with general anaesthesia soon after receiving chemotherapy; occasionally such treatment can be given during surgery. Therefore, it is worthwhile and prudent to understand the pathophysiology of cancer and consider the pharmacological interactions between anticancer and anaesthetic drugs. Anti-cancer chemotherapeutic drugs may cause generalized and specific organ toxicities and may also give rise to various unpredictable or life-threatening peri-operative complications, rendering a detailed pre-operative assessment of patients with previous chemotherapy mandatory. Thus, special consideration and understanding of the cancer patient's anaesthesia-related needs will result in superior patient care and outcomes.

## References

[1] Jessup JM, McGinnis LS, Steelo GO (1996). The national cancer database: Report on colon cancer. Cancer.

[2] Stephen P, Fischer (1998). Preoperative evaluation of the cancer patient; Anesthesia Clinics of North America.

[3] Foley JF, Foley J, Vose J, Armitage JA (1999). Complications of chemotherapy agents. Current Therapy in Cancer.

[4] Burrows FA, Hickey PR, Colan S (1985). Perioperative complications in patients with anthracycline chemotherapeutic agents. Can Anaesth Soc J.

[5] Lahtinen R, Kuikka J, Nousianinen T (1991). Cardiotoxicity of epirubicin and doxorubicin: A double-blind randomized study. Eur J Haematol.

[6] Lekakis J, Vassilopoulos N, Psichoyiou H (1991). Doxorubicin cardiotoxicity detected by indium 111 myosin-specific imaging. Eur J Nucl Med.

[7] Weesner KM, Bledsoe M, Chauvenet A (1991). Exercise echocardiography in the detection of anthracycline cardiotoxicity. Cancer.

[8] Lewkow LM, Hooker JL, Movahed A (1992). Cardiac complications of intensive dose mitoxantrone and cyclophosphamide with autologous bone marrow transplantation in metastatic breast cancer. Int J Cardiol.

[9] Solley GO, Maldonado JE, Gleich GJ (1976). Endomyocardiopathy with eosinophilia. Mayo Clin Proc.

[10] Drzewoski J, Kasznicki J (1992). Cardiotoxicity of antineoplastic drugs. Acta Haematol Pol.

[11] Verweij J (1996). Mitomycins. Cancer Chemother Biol Response Modif.

[12] Huettemann E, Junker T, Chatzinikolaou KP (2004). The influence of anthracycline therapy on cardiac function during anesthesia. Anesth Analg.

[13] Steinherz LJ, Steinherz PG, Tan CT (1991). Cardiac toxicity 4 to 20 years after completing anthracycline therapy. JAMA.

[14] Burrows FA, Hickey PR, Colan S (1985). Perioperative complications in patients with anthracycline chemotherapeutic agents. Can Anaesth Soc J.

[15] Cortes JE, Pazdur R (1995). Docetaxel. J Clin Oncol.

[16] Steinberg JS, Cohen AJ, Wasserman AG, Cohen P, Ross AM (1987). Acute arrhythmogenecity of doxorubicin administration. Cancer.

[17] Bristow MR, Billingham ME, Mason JW, Daniels JR (1978). Clinical spectrum of anthracycline antibiotic cardiotoxicity. Cancer Treat Rep.

[18] Lefor AT (1999). Perioperative management of the patient with cancer. Chest.

[19] Praga C, Beretta G, Vigo PL, Pollini C, Bonadonna G, Canetta R (1979). Adriamycin cardiotoxicity: A survey of 1273 patients. Cancer Treat Rep.

[20] Von Hoff DD, Layward MW, Basa P, Davis HL, Von Hoff AL, Rozencweig M (1979). Risk factors for doxorubicin–induced congestive heart failure. Ann Intern Med.

[21] Shan K, Lincoff AM, Young JB (1996). Anthracycline-induced cardiotoxicity. Ann Intern Med.

[22] Steinherz LJ, Steinherz PG, Tan CT, Heller G, Murphy ML (1991). Cardiac toxicity 4 to 20 years after completing anthracycline therapy. JAMA.

[23] Lipshultz SE, Colan SD, Gelber RD, Perez Atayde AR, Sallan SE, Sanders SP (1991). Late cardiac effects of doxorubicin therapy for acute lymphoblastic leukemia in childhood. N Engl J Med.

[24] Ganz WI, Sridhar KS, Ganz SS, Chakko S, Serafini A (1996). Review of tests for monitoring Doxorubicin-induced Cardiomyopathy. Oncology.

[25] Varon J (1995). Acute respiratory distress syndrome in the postoperative cancer patient. The Cancer Bulletin.

[26] Dumont P, Wh JM, Hentz JG (1995). Respiratory complications after surgical treatment of esophageal cancer: A study of 309 patients according to the type of resection. Eur J Cardiothorac Surg.

[27] Epner De, White F, Krasnoff M (1996). Outcome of mechanical ventilation for adults with hematologic malignancy. J Invest Med.

[28] Randle CJ, Frankel LR, Amylon MD (1996). Identifying early predictors of mortality in pediatric patients with acute leukemia and pneumonia. Chest.

[29] Lombara CM, Churg A, Winokur S (1987). Pulmonary veno-occlusive disease following therapy for malignant neoplasms. Chest.

[30] Meysman M, Schoors DF, Reynaert H Respiratory failure with diffuse patchy lung infiltrates: An unusual presentation of squamous cell carcinoma. Thorax.

[31] Williams DM, Krick JA, Remington JS (1976). Pulmonary infection in the compromised host: Oncol Biol Part I. Am Rev Respir Dis.

[32] Waid-Jones M, Coursin DB (1991). Perioperative considerations for patients treated with bleomycin. Chest.

[33] Rosenow EC, Myers JL, Swensen SJ (1992). Drug-induced pulmonary disease: An update. Chest.

[34] Goldiner PL, Schweizer O (1979). The hazards of anesthesia and surgery in Bleomycin-treated patients. Seminars in Oncology.

[35] Goldiner PL, Carlon GC, Cvitkovic E, Schweizer O, Howland W (1978). Factors influencing postoperative morbidity and mortality in patients treated with bleomycin. Britsh Medical Journal.

[36] LaMantia KR, Glick JH, Marshall BE (1984). Supplemental oxygen does not cause respiratory failure in Bleomycin-treated surgical patients. Anesthesiology.

[37] Donat SM, Levy DA (1998). Bleomycin associated pulmonary toxicity: is perioperative oxygen restriction necessary?. The Journal of Urology.

[38] Madias NE, Harrinton JT (1978). Platinum nephrotoxicity. Am J Med.

[39] Fjeldberg P, Sorensen J, Helkjaer PE (1986). The long term effects of cisplatin on renal function. Cancer.

[40] El-Badawi MG, Abdalla MA, Bahakim HM, Fadel RA (1996). Nephrotoxicity of low-dose methotrexate in guinea pigs: an ultrastructural study. Nephron.

[41] Weiss HD, Walker MD, Wiernik PH (1974). Neurotoxicity of commonly used antineoplastic agents. NEJM.

[42] Kedar A, Cohen ME, Freeman AI (1978). Peripheral neuropathy as a complication of cis-Dichlorodiammine-platinum treatment: a case report. Cancer treatment reports.

[43] Siemsen JK, Meister L (1963). Bronchogenic carcinoma associated with severe orthostatic hypotension. Ann Int Med.

[44] Huettemann, Egbert Sakka, Samir G (2005). Anaesthesia and anti-cancer chemotherapeutic drugs. Anaesthesia and medical disease. Current Opinion in Anaesthesiology.

[45] Heyman MR, Lefor AT (1996). Cancer and therapy-related hematologic abnormalities. Surgical problems affecting the patient with cancer.

[46] Anderson RJ, Chung HM, Kluge R (1985). Hyponatremia: A prospective analysis of its epidemiologyand the pathogenetic role of vasopressin. Ann Intern Med.

[47] Fleming DR, Doukas MA (1992). Acute tumor lysis syndrome in hematologic mahgnancies. Leuk Lymphoma.

[48] Jones DP, Mahmoud H, chesney RW (1995). Tumor lysis syndrome: Pathogenesis and management. Pediatr NephroI.

[49] Van der Hoven B, Thunnissen PL, Sizoo W (1992). Tumour lysis syndrome in haematological malignancies. Neth J Med.

[50] Scha¨ffer MR, Barbul A, Lefor AT (1996). Chemotherapy and wound healing. Surgical problems affecting the patient with cancer.

[51] Mathes DD, Bogdonoff DL, Lefor AT (1996). Preoperative evaluation of the cancer patient. Surgical problems affecting the patient with cancer.

[52] Klein DS, Wilds PR (1983). Pulmonary toxicity of antineoplastic agents: anaesthetic and postoperative implications. Can Anaesth Soc J.

[53] Zsigmond EK, Robins G (1972). The effect of a series of anticancer drugs on plasma cholinesterase activity. Can Anaesth Soc J.

[54] Frenia ML, Long KS (1992). Methotrezate and NSAID interactions. Ann Pharmacother.

